# Supplementary material for: Self-reported and measured anthropometric variables in association with cardiometabolic markers: A Danish cohort study
Source: PLoS One. 2023 Jul 27;18(7):e0279795. doi: 10.1371/journal.pone.0279795 (PMC10374072; doi:10.1371/journal.pone.0279795)
Supplement: S1 Table — (DOCX) [file pone.0279795.s001.docx]

S1 Table. Misreporting on anthropometric variables according to participant characteristics

|  | **Height Diff*** | | **Weight Diff*** | | **BMI Diff*** | | **WC Diff*** | | **WHtR Diff*** | |
| --- | --- | --- | --- | --- | --- | --- | --- | --- | --- | --- |
|  | **Mean** | **sd** | **mean** | **sd** | **mean** | **sd** | **mean** | **sd** | **mean** | **sd** |
| **Sex** |  |  |  |  |  |  |  |  |  |  |
| Male | 1.21 | 1.81 | -0.11 | 2.31 | -0.38 | 0.89 | -0.12 | 6.56 | -0.00 | 0.04 |
| Female | 0.97 | 1.62 | -0.47 | 2.01 | -0.45 | 0.88 | -0.25 | 5.67 | -0.00 | 0.03 |
| **Age groups** | |  |  |  |  |  |  |  |  |  |
| <=25 | 0.98 | 1.68 | -0.10 | 2.65 | -0.30 | 0.95 | -0.67 | 5.94 | -0.01 | 0.03 |
| 26-35 | 0.78 | 1.75 | -0.06 | 2.28 | -0.24 | 0.92 | -0.61 | 5.96 | -0.01 | 0.03 |
| 36-45 | 0.85 | 1.58 | -0.25 | 2.00 | -0.33 | 0.82 | 0.61 | 5.80 | 0.00 | 0.03 |
| 46-55 | 1.07 | 1.65 | -0.45 | 2.06 | -0.47 | 0.87 | 0.12 | 5.88 | -0.00 | 0.03 |
| 56-65 | 1.44 | 1.81 | -0.40 | 1.92 | -0.57 | 0.86 | -0.78 | 6.49 | -0.01 | 0.04 |
| >65 | 2.13 | 1.94 | -0.34 | 1.86 | -0.74 | 0.83 | -1.27 | 5.38 | -0.01 | 0.03 |
| **Smoking** |  |  |  |  |  |  |  |  |  |  |
| Current | 1.15 | 1.76 | -0.19 | 2.23 | -0.40 | 0.90 | -0.51 | 6.37 | -0.01 | 0.04 |
| Former | 1.14 | 1.67 | -0.42 | 2.02 | -0.49 | 0.87 | -0.20 | 5.86 | -0.00 | 0.03 |
| Never | 1.00 | 1.71 | -0.30 | 2.19 | -0.40 | 0.89 | -0.11 | 6.04 | -0.00 | 0.04 |
| **BMI**  **Classification** |  |  |  |  |  |  |  |  |  |  |
| Underweight | 0.70 | 1.55 | 0.98 | 4.26 | 0.18 | 1.22 | 1.28 | 4.14 | 0.01 | 0.02 |
| Normal weight | 0.84 | 1.62 | 0.00 | 1.81 | -0.22 | 0.72 | 0.56 | 5.16 | 0.00 | 0.03 |
| Overweight | 1.28 | 1.71 | -0.63 | 2.08 | -0.60 | 0.85 | -0.85 | 6.45 | -0.01 | 0.04 |
| Obese | 1.60 | 1.95 | -1.12 | 2.86 | -0.97 | 1.22 | -2.18 | 8.01 | -0.02 | 0.05 |

Sd, standard deviation; BMI, body mass index; WC, waist circumference; WHtR, waist-to-height ratio

Diff, difference, which was calculated by subtracting measured from self-reported values
