# Supplementary material for: Self-reported and measured anthropometric variables in association with cardiometabolic markers: A Danish cohort study
Source: PLoS One. 2023 Jul 27;18(7):e0279795. doi: 10.1371/journal.pone.0279795 (PMC10374072; doi:10.1371/journal.pone.0279795)
Supplement: S2 Table — (DOCX) [file pone.0279795.s002.docx]

S2 Table. Factors associated with misreporting on anthropometric variables in multivariable regression models

|  | **Height Diff*** | | | **Weight Diff*** | | | **BMI Diff*** | | | **WC Diff*** | | | **WHtR Diff*** | | |
| --- | --- | --- | --- | --- | --- | --- | --- | --- | --- | --- | --- | --- | --- | --- | --- |
|  | coef | 95%CI | | coef | 95%CI | | coef | 95%CI | | coef | 95%CI | | coef | 95%CI | |
| **Age groups** | |  |  |  |  |  |  |  |  |  |  |  |  |  |  |
| <=25 | ref |  |  |  |  |  |  |  |  |  |  |  |  |  |  |
|  |  |  |  |  |  |  |  |  |  |  |  |  |  |  |  |
| 26-35 | -0.28 | -0.34 | 0.21 | 0.15 | 0.07 | 0.23 | 0.13 | 0.09 | 0.16 | 0.31 | 0.00 | 0.62 | 0.00 | 0.00 | 0.00 |
|  |  |  |  |  |  |  |  |  |  |  |  |  |  |  |  |
| 36-45 | -0.24 | -0.31 | -0.18 | 0.05 | -0.04 | 0.13 | 0.08 | 0.05 | 0.11 | 1.68 | 1.38 | 1.98 | 0.01 | 0.01 | 0.01 |
|  |  |  |  |  |  |  |  |  |  |  |  |  |  |  |  |
| 46-55 | -0.07 | -0.12 | -0.01 | -0.07 | -0.14 | 0.00 | -0.01 | -0.04 | 0.02 | 1.36 | 1.10 | 1.62 | 0.01 | 0.01 | 0.01 |
|  |  |  |  |  |  |  |  |  |  |  |  |  |  |  |  |
| 56-65 | 0.26 | 0.20 | 0.32 | 0.00 | -0.08 | 0.08 | -0.09 | -0.12 | -0.06 | 0.54 | 0.26 | 0.82 | 0.00 | 0.00 | 0.00 |
|  |  |  |  |  |  |  |  |  |  |  |  |  |  |  |  |
| >65 | 0.85 | 0.67 | 1.04 | -0.03 | -0.27 | 0.21 | -0.26 | -0.36 | -0.17 | 0.17 | -0.57 | 0.91 | 0.00 | -0.01 | 0.00 |
| **Sex** |  |  |  |  |  |  |  |  |  |  |  |  |  |  |  |
| Female | ref |  |  |  |  |  |  |  |  |  |  |  |  |  |  |
| Male | 0.14 | 0.11 | 0.18 | 0.51 | 0.47 | 0.56 | 0.17 | 0.15 | 0.18 | 0.48 | 0.33 | 0.63 | 0.00 | 0.00 | 0.00 |
| **Smoking** |  |  |  |  |  |  |  |  |  |  |  |  |  |  |  |
| Never | ref |  |  |  |  |  |  |  |  |  |  |  |  |  |  |
| Current | 0.14 | 0.09 | 0.18 | 0.12 | 0.06 | 0.18 | 0.00 | -0.02 | 0.02 | -0.32 | -0.52 | -0.11 | 0.00 | 0.00 | 0.00 |
| Former | 0.05 | 0.01 | 0.09 | 0.01 | -0.04 | 0.06 | -0.01 | -0.03 | 0.01 | -0.01 | -0.18 | 0.16 | 0.00 | 0.00 | 0.00 |
| **BMI classification** | | | | | | | | | | | | | | | |
| Normal weight | Ref |  |  |  |  |  |  |  |  |  |  |  |  |  |  |
| Underweight | -0.10 | -0.23 | 0.04 | -1.06 | 0.89 | 1.23 | 0.41 | 0.35 | 0.48 | 1.09 | 0.52 | 1.66 | 0.01 | 0.00 | 0.01 |
| Overweight | 0.36 | 0.32 | 0.40 | -0.72 | -0.77 | -0.67 | -0.39 | -0.41 | -0.37 | -1.60 | -1.77 | -1.44 | -0.01 | -0.01 | -0.01 |
| Obese | 0.69 | 0.63 | 0.74 | -1.16 | -1.23 | -1.09 | -0.75 | -0.78 | -0.72 | -2.91 | -3.15 | -2.67 | -0.02 | -0.02 | -0.02 |
| *n* | 39,365 |  |  | 37,700 |  |  | 37,627 |  |  | 26,053 |  |  | 25,996 |  |  |
| adj. *R*^2^ | 0.04 |  |  | 0.06 |  |  | 0.11 |  |  | 0.04 |  |  | 0.05 |  |  |

BMI, body mass index; WC, waist circumference; WHtR, waist-to-height ratio; *R*^2^, R-squared; ref, reference

Diff, difference

*Difference was calculated by subtracting measured from self-reported values
