# Supplementary material for: Self-reported and measured anthropometric variables in association with cardiometabolic markers: A Danish cohort study
Source: PLoS One. 2023 Jul 27;18(7):e0279795. doi: 10.1371/journal.pone.0279795 (PMC10374072; doi:10.1371/journal.pone.0279795)
Supplement: S3 Table — (DOCX) [file pone.0279795.s003.docx]

S3 Table. BMI Classification Based on measured and self-reported BMI

|  | **Total** | | | | **Male** | | | | **Female** | | | |
| --- | --- | --- | --- | --- | --- | --- | --- | --- | --- | --- | --- | --- |
| **BMI** | **Measure BMI** | | **Self-reported BMI** | | **Measure BMI** | | **Self-reported BMI** | | **Measure BMI** | | **Self-reported BMI** | |
| **Classification*** | **%** | n | **%** | n | **%** | n | **%** | n | **%** | n | **%** | n |
| Underweight | 1.7 | 635 | 1.9 | 721 | 0.7 | 104 | 0.7 | 107 | 2.5 | 531 | 2.9 | 614 |
| Normal | 54.1 | 20,378 | 58.6 | 22,075 | 44.7 | 7,198 | 49.5 | 7,976 | 61.2 | 1,318 | 65.5 | 14,099 |
| Overweight | 32.5 | 12,230 | 29.7 | 11,190 | 42.1 | 6,794 | 39.7 | 6,406 | 25.2 | 5,436 | 22.2 | 4,784 |
| Obese | 11.7 | 4,417 | 9.8 | 3,674 | 12.6 | 2,025 | 10.1 | 1,632 | 11.1 | 2,392 | 9.5 | 2,042 |
| Total | 100.0 | 37,660 | 100.0 | 37,660 | 100.0 | 16,121 | 100.0 | 16,121 | 100.0 | 21,539 | 100.0 | 21,539 |

*BMI was calculated as weight (kg) divided by height squared (m^2^) and subsequently categorized into 4 groups according to World Health Organization criteria: Underweight (<18.5 kg/ m^2^); Normal (18.5 to <24.9 kg/ m^2^); Overweight (25 to <29.9 kg/ m^2^); Obese (>=30 kg/m^2^)
