# Supplementary material for: Self-reported and measured anthropometric variables in association with cardiometabolic markers: A Danish cohort study
Source: PLoS One. 2023 Jul 27;18(7):e0279795. doi: 10.1371/journal.pone.0279795 (PMC10374072; doi:10.1371/journal.pone.0279795)
Supplement: S4 Table — (DOCX) [file pone.0279795.s004.docx]

S4 Table. Cross-classification of measured and self-reported BMI classification

|  | **Measured BMI** | | | |
| --- | --- | --- | --- | --- |
| **Self-reported BMI** | **Underweight** | **Normal** | **Overweight** | **Obese** |
| Underweight | 76.54% | 1.13% | 0.03% | 0.00% |
| Normal | 23.31% | 96.55% | 18.38% | 0.09% |
| Overweight | 0.00% | 2.27% | 80.39% | 20.26% |
| Obese | 0.16% | 0.04% | 1.19% | 79.65% |

*BMI was calculated as weight (kg) divided by height squared (m^2^) and subsequently categorized into 4 groups according to World Health Organization criteria: Underweight(<18.5 kg/ m^2^) ; Normal(18.5 to <24.9 kg/ m^2^); Overweight (25 to <29.9 kg/ m^2^); Obese(>=30 kg/m^2^)
