# Supplementary material for: Self-reported and measured anthropometric variables in association with cardiometabolic markers: A Danish cohort study
Source: PLoS One. 2023 Jul 27;18(7):e0279795. doi: 10.1371/journal.pone.0279795 (PMC10374072; doi:10.1371/journal.pone.0279795)
Supplement: S5 Table — (DOCX) [file pone.0279795.s005.docx]

S5 Table. Agreement between self-reported indices compared to measured indices

|  | **BMI categories*** | | | **BMI categories^Ø^** | | |  | | | **Self-reported obesity with measured central obesity** | | | **Measured obesity with measured central obesity** | | |
| --- | --- | --- | --- | --- | --- | --- | --- | --- | --- | --- | --- | --- | --- | --- | --- |
|  | **(WHO criteria)** | | | **(Normal versus owob)** | | | **WC categories**** | | |  |  |  |  |  |  |
|  | Coef. | 95% CI | | Coef. | 95% CI | | Coef. | 95% CI | | Coef. | 95% CI | | Coef. | 95% CI | |
| Cohen/Conger's Kappa | 0.81 | 0.80 | 0.81 | 0.86 | 0.85 | 0.86 | 0.72 | 0.71 | 0.73 | 0.47 | 0.46 | 0.48 | 0.53 | 0.52 | 0.54 |
| Scott/Fleiss' Pi | 0.81 | 0.80 | 0.82 | 0.86 | 0.85 | 0.87 | 0.72 | 0.71 | 0.74 | 0.45 | 0.44 | 0.47 | 0.52 | 0.51 | 0.53 |
| Gwet's AC | 0.86 | 0.86 | 0.87 | 0.97 | 0.96 | 0.97 | 0.84 | 0.83 | 0.85 | 0.77 | 0.77 | 0.78 | 0.79 | 0.78 | 0.80 |

*BMI: 1"Underweight" 2"Normal" 3"Overweight" 4"Obese" according to WHO criteria, self-reported BMI categories compared to measured BMI categories

Coef, coefficient

^ø^BMI: Normal versus overweight/obese, self-reported BMI categories compared to measured BMI categories

**WC: adiposity if >88cm for women and >102cm for men, self-reported WC adiposity compared to measured WC adiposity
