# Supplementary material for: Self-reported and measured anthropometric variables in association with cardiometabolic markers: A Danish cohort study
Source: PLoS One. 2023 Jul 27;18(7):e0279795. doi: 10.1371/journal.pone.0279795 (PMC10374072; doi:10.1371/journal.pone.0279795)
Supplement: S6 Table — (DOCX) [file pone.0279795.s006.docx]

S6 Table. Association between self-reported and measured anthropometric variables and CVD biomarkers* (Standarized)

|  |  | **Measured BMI** | | **Self-reported BMI** | | **Measured WC** | | **Self-reported WC** | | **Measured WHtR** | | **Self-reported WHtR** | |
| --- | --- | --- | --- | --- | --- | --- | --- | --- | --- | --- | --- | --- | --- |
|  |  | beta | se | beta | se | beta | se | beta | se | beta | se | beta | se |
| Crude models | TG(mmol/L)* | 0.40 | 0.01 | 0.39 | 0.00 | 0.49 | 0.00 | 0.44 | 0.01 | 0.45 | 0.00 | 0.41 | 0.01 |
|  | Cholesterol | 0.20 | 0.01 | 0.19 | 0.01 | 0.26 | 0.01 | 0.23 | 0.01 | 0.29 | 0.01 | 0.25 | 0.01 |
|  | (mmol/L) |  |  |  |  |  |  |  |  |  |  |  |  |
|  | HDL(mmol/L) | -0.32 | 0.01 | -0.34 | 0.00 | -0.38 | 0.00 | -0.37 | 0.01 | -0.30 | 0.01 | -0.29 | 0.01 |
|  | LDL(mmol/L) | 0.24 | 0.01 | 0.23 | 0.01 | 0.31 | 0.00 | 0.28 | 0.01 | 0.31 | 0.00 | 0.28 | 0.01 |
|  | HbA1  (mmol/mol)* | 0.30 | 0.01 | 0.30 | 0.01 | 0.36 | 0.00 | 0.34 | 0.01 | 0.36 | 0.00 | 0.33 | 0.01 |
|  | CRP (mg/L) * | 0.37 | 0.01 | 0.36 | 0.01 | 0.32 | 0.01 | 0.26 | 0.01 | 0.37 | 0.00 | 0.31 | 0.01 |
|  | Creatinine  (μmol/L)* | 0.12 | 0.01 | 0.13 | 0.01 | 0.24 | 0.01 | 0.26 | 0.01 | 0.06 | 0.01 | 0.09 | 0.01 |
|  | SBP (mmHg) | 0.32 | 0.01 | 0.31 | 0.01 | 0.42 | 0.00 | 0.38 | 0.01 | 0.35 | 0.00 | 0.31 | 0.01 |
|  | DBP(mmHg) | 0.34 | 0.01 | 0.33 | 0.00 | 0.41 | 0.00 | 0.36 | 0.01 | 0.39 | 0.00 | 0.34 | 0.01 |
| Adjusted models^Ø^ | TG(mmol/L)* | 0.34 | 0.01 | 0.32 | 0.01 | 0.43 | 0.01 | 0.37 | 0.01 | 0.41 | 0.01 | 0.35 | 0.01 |
|  | Cholesterol | 0.09 | 0.01 | 0.08 | 0.01 | 0.11 | 0.01 | 0.09 | 0.01 | 0.12 | 0.01 | 0.10 | 0.01 |
|  | (mmol/L) |  |  |  |  |  |  |  |  |  |  |  |  |
|  | HDL(mmol/L) | -0.34 | 0.01 | -0.34 | 0.01 | -0.41 | 0.01 | -0.38 | 0.01 | -0.39 | 0.01 | -0.36 | 0.01 |
|  | LDL(mmol/L) | 0.14 | 0.01 | 0.14 | 0.01 | 0.17 | 0.01 | 0.15 | 0.01 | 0.17 | 0.01 | 0.15 | 0.01 |
|  | HbA1c(mmol/mol)* | 0.21 | 0.01 | 0.22 | 0.01 | 0.26 | 0.01 | 0.23 | 0.01 | 0.24 | 0.01 | 0.22 | 0.01 |
|  | CRP (mg/L) * | 0.40 | 0.01 | 0.39 | 0.01 | 0.45 | 0.01 | 0.38 | 0.01 | 0.44 | 0.01 | 0.36 | 0.01 |
|  | Creatinine(μmol/L)* | 0.01 | 0.00 | 0.01 | 0.00 | -0.02 | 0.01 | -0.02 | 0.01 | -0.04 | 0.00 | -0.04 | 0.01 |
|  | SBP (mmHg) | 0.20 | 0.00 | 0.18 | 0.01 | 0.20 | 0.01 | 0.16 | 0.01 | 0.19 | 0.01 | 0.15 | 0.01 |
|  | DBP(mmHg) | 0.24 | 0.01 | 0.23 | 0.01 | 0.27 | 0.01 | 0.22 | 0.01 | 0.26 | 0.01 | 0.21 | 0.01 |

TG triglycerides; HDL, high-density lipoprotein; LDL, low-density lipoprotein; HbA1c, hemoglobin A1c; CRP, C-reactive Protein; SBP, systolic blood pressure; DBP, diastolic blood pressure; BMI, body mass index; WC, waist circumference; WHtR, waist-to-height ratio; CVD, cardiovascular disease

^Ø^Models were adjusted for age, sex, and smoking *values were log-transformed

Standardized β coefficient
