# Supplementary material for: Self-reported and measured anthropometric variables in association with cardiometabolic markers: A Danish cohort study
Source: PLoS One. 2023 Jul 27;18(7):e0279795. doi: 10.1371/journal.pone.0279795 (PMC10374072; doi:10.1371/journal.pone.0279795)
Supplement: S7 Table — (DOCX) [file pone.0279795.s007.docx]

S7 Table. Association between self-reported and measured anthropometric variables and CVD biomarkers* (Unstandarized)

|  |  | **Measured BMI** | | **Self-reported BMI** | | **Measured WC** | | **Self-reported WC** | | **Measured WHtR** | | **Self-reported WHtR** | |
| --- | --- | --- | --- | --- | --- | --- | --- | --- | --- | --- | --- | --- | --- |
|  |  | coef | se | coef | se | coef | se | coef | se | coef | se | coef | se |
| Crude models | TG(mmol/L)* | 0.05 | 0.00 | 0.05 | 0.00 | 0.02 | 0.00 | 0.02 | 0.00 | 3.42 | -0.03 | 3.20 | -0.04 |
|  | Cholesterol  (mmol/L) | 0.05 | 0.00 | 0.05 | 0.00 | 0.02 | 0.00 | 0.02 | 0.00 | 4.13 | -0.07 | 3.76 | -0.09 |
|  | HDL(mmol/L) | -0.03 | 0.00 | -0.04 | 0.00 | -0.01 | 0.00 | -0.01 | 0.00 | -1.89 | -0.03 | -1.93 | -0.04 |
|  | LDL(mmol/L) | 0.05 | 0.00 | 0.05 | 0.00 | 0.02 | 0.00 | 0.02 | 0.00 | 4.03 | -0.06 | 3.80 | -0.08 |
|  | HbA1c(mmol/mol)* | 0.01 | 0.00 | 0.01 | 0.00 | 0.00 | 0.00 | 0.00 | 0.00 | 0.40 | -0.01 | 0.39 | -0.01 |
|  | CRP (mg/L) * | 0.09 | 0.00 | 0.09 | 0.00 | 0.03 | 0.00 | 0.02 | 0.00 | 5.30 | -0.07 | 4.62 | -0.09 |
|  | Creatinine(μmol/L)* | 0.01 | 0.00 | 0.01 | 0.00 | 0.00 | 0.00 | 0.00 | 0.00 | 0.17 | -0.01 | 0.24 | -0.02 |
|  | SBP (mmHg) | 1.26 | -0.02 | 1.28 | -0.02 | 0.54 | -0.01 | 0.51 | -0.01 | 82.01 | -1.12 | 77.13 | -1.47 |
|  | DBP(mmHg) | 0.91 | -0.01 | 0.93 | -0.01 | 0.36 | 0.00 | 0.33 | -0.01 | 62.31 | -0.75 | 56.35 | -0.98 |
| Adjusted models^Ø^ | TG(mmol/L) * | 0.04 | 0.00 | 0.04 | 0.00 | 0.02 | 0.00 | 0.02 | 0.00 | 3.10 | -0.04 | 2.75 | -0.05 |
|  | Cholesterol  (mmol/L) | 0.02 | 0.00 | 0.02 | 0.00 | 0.01 | 0.00 | 0.01 | 0.00 | 1.76 | -0.07 | 1.51 | -0.09 |
|  | HDL(mmol/L) | -0.03 | 0.00 | -0.04 | 0.00 | -0.01 | 0.00 | -0.01 | 0.00 | -2.44 | -0.03 | -2.34 | -0.04 |
|  | LDL(mmol/L) | 0.03 | 0.00 | 0.03 | 0.00 | 0.01 | 0.00 | 0.01 | 0.00 | 2.25 | -0.07 | 2.06 | -0.08 |
|  | HbA1c(mmol/mol)* | 0.00 | 0.00 | 0.00 | 0.00 | 0.00 | 0.00 | 0.00 | 0.00 | 0.27 | -0.01 | 0.25 | -0.01 |
|  | CRP (mg/L) * | 0.10 | 0.00 | 0.10 | 0.00 | 0.04 | 0.00 | 0.03 | 0.00 | 6.33 | -0.08 | 5.44 | -0.10 |
|  | Creatinine(μmol/L)* | 0.00 | 0.00 | 0.00 | 0.00 | 0.00 | 0.00 | 0.00 | 0.00 | -0.11 | -0.01 | -0.10 | -0.01 |
|  | SBP (mmHg) | 0.76 | -0.02 | 0.74 | -0.02 | 0.26 | -0.01 | 0.21 | -0.01 | 45.30 | -1.12 | 37.08 | -1.45 |
|  | DBP(mmHg) | 0.64 | -0.01 | 0.64 | -0.01 | 0.24 | -0.01 | 0.20 | -0.01 | 41.75 | -0.79 | 34.90 | -1.02 |

^Ø^Adjusted for age, sex, and smoking *log-transformed

TG triglycerides; HDL, high-density lipoprotein; LDL, low-density lipoprotein; HbA1c, hemoglobin A1c; CRP, C-reactive Protein; SBP, systolic blood pressure; DBP, diastolic blood pressure; BMI, body mass index; WC, waist circumference; WHtR, waist-to-height ratio; CVD, cardiovascular disease

^Ø^Adjusted for age, sex, and smoking

*values were log-transformed
