# Supplementary material for: Self-reported and measured anthropometric variables in association with cardiometabolic markers: A Danish cohort study
Source: PLoS One. 2023 Jul 27;18(7):e0279795. doi: 10.1371/journal.pone.0279795 (PMC10374072; doi:10.1371/journal.pone.0279795)
Supplement: S8 Table — (DOCX) [file pone.0279795.s008.docx]

S8 Table. Association between self-reported and measured anthropometric variables and CVD biomarkers stratified by age

|  |  | **Measured BMI** | | **Self-reported BMI** | | **Measured WC** | | **Self-reported WC** | | **Measured WHtR** | | **Self-reported WHtR** | |
| --- | --- | --- | --- | --- | --- | --- | --- | --- | --- | --- | --- | --- | --- |
| **Dependent variables** | | beta | se | beta | se | beta | se | beta | se | beta | se | beta | se |
| TG(mmol/L)* | age<=45 | 0.31 | 0.00 | 0.29 | 0.00 | 0.37 | 0.00 | 0.32 | 0.00 | 0.35 | 0.58 | 0.30 | 0.79 |
|  | age46-55 | 0.37 | 0.00 | 0.36 | 0.00 | 0.45 | 0.00 | 0.39 | 0.00 | 0.42 | 0.55 | 0.37 | 0.70 |
|  | age>55 | 0.34 | 0.00 | 0.33 | 0.00 | 0.41 | 0.00 | 0.36 | 0.00 | 0.38 | 0.76 | 0.34 | 0.94 |
| Cholesterol | age<=45 | 0.22 | 0.00 | 0.22 | 0.00 | 0.28 | 0.00 | 0.25 | 0.00 | 0.28 | 1.10 | 0.24 | 1.46 |
| (mmol/L) | age46-55 | 0.10 | 0.00 | 0.10 | 0.00 | 0.13 | 0.00 | 0.12 | 0.00 | 0.14 | 1.12 | 0.13 | 1.40 |
|  | age>55 | -0.01 | 0.00 | -0.02 | 0.00 | 0.01 | 0.00 | 0.01 | 0.00 | 0.01 | 0.00 | 0.01 | 0.00 |
| HDL(mmol/L) | age<=45 | -0.25 | 0.00 | -0.25 | 0.00 | -0.28 | 0.00 | -0.25 | 0.00 | -0.26 | 0.46 | -0.23 | 0.62 |
|  | age46-55 | -0.36 | 0.00 | -0.36 | 0.00 | -0.41 | 0.00 | -0.38 | 0.00 | -0.38 | 0.47 | -0.35 | 0.59 |
|  | age>55 | -0.34 | 0.00 | -0.35 | 0.00 | -0.39 | 0.00 | -0.35 | 0.00 | -0.36 | 0.68 | -0.32 | 0.84 |
| LDL(mmol/L) | age<=45 | 0.26 | 0.00 | 0.26 | 0.00 | 0.32 | 0.00 | 0.29 | 0.00 | 0.31 | 0.98 | 0.28 | 1.30 |
|  | age46-55 | 0.15 | 0.00 | 0.15 | 0.00 | 0.19 | 0.00 | 0.18 | 0.00 | 0.19 | 1.01 | 0.18 | 1.27 |
|  | age>55 | 0.04 | 0.00 | 0.04 | 0.00 | 0.06 | 0.00 | 0.06 | 0.00 | 0.05 | 2.55 | 0.05 | 2.80 |
| HbA1c(mmol/mol)* | age<=45 | 0.18 | 0.00 | 0.19 | 0.00 | 0.21 | 0.00 | 0.20 | 0.00 | 0.19 | 0.00 | 0.18 | 0.00 |
|  | age46-55 | 0.26 | 0.00 | 0.26 | 0.00 | 0.30 | 0.00 | 0.28 | 0.00 | 0.28 | 0.00 | 0.25 | 0.00 |
|  | age>55 | 0.28 | 0.00 | 0.28 | 0.00 | 0.31 | 0.00 | 0.29 | 0.00 | 0.30 | 0.00 | 0.27 | 0.00 |
| CRP (mg/L)* | age<=45 | 0.34 | 0.00 | 0.33 | 0.00 | 0.34 | 0.00 | 0.27 | 0.00 | 0.32 | 1.36 | 0.26 | 1.85 |
|  | age46-55 | 0.43 | 0.00 | 0.43 | 0.00 | 0.47 | 0.00 | 0.41 | 0.00 | 0.44 | 1.09 | 0.38 | 1.40 |
|  | age>55 | 0.41 | 0.00 | 0.41 | 0.00 | 0.45 | 0.00 | 0.37 | 0.00 | 0.43 | 1.51 | 0.36 | 1.87 |
| Creatinine(μmol/L)* | age<=45 | 0.03 | 0.00 | 0.03 | 0.00 | -0.01 | 0.00 | -0.01 | 0.00 | -0.03 | 0.00 | -0.02 | 0.00 |
|  | age46-55 | 0.00 | 0.00 | 0.00 | 0.00 | -0.03 | 0.00 | -0.02 | 0.00 | -0.04 | 0.00 | -0.03 | 0.00 |
|  | age>55 | 0.01 | 0.00 | 0.01 | 0.00 | -0.01 | 0.00 | -0.02 | 0.00 | -0.03 | 0.00 | -0.03 | 0.00 |
| SBP(mmHg) | age<=45 | 0.24 | 0.02 | 0.23 | 0.02 | 0.24 | 0.01 | 0.21 | 0.01 | 0.21 | 1.41 | 0.18 | 1.90 |
|  | age46-55 | 0.23 | 0.03 | 0.22 | 0.03 | 0.24 | 0.01 | 0.20 | 0.01 | 0.23 | 1.82 | 0.18 | 2.30 |
|  | age>55 | 0.17 | 0.04 | 0.15 | 0.05 | 0.15 | 0.02 | 0.13 | 0.02 | 0.16 | 2.74 | 0.13 | 3.31 |
| DBP(mmHg) | age<=45 | 0.30 | 0.02 | 0.29 | 0.02 | 0.34 | 0.01 | 0.29 | 0.01 | 0.32 | 1.16 | 0.27 | 1.57 |
|  | age46-55 | 0.28 | 0.02 | 0.27 | 0.02 | 0.30 | 0.01 | 0.25 | 0.01 | 0.29 | 1.27 | 0.24 | 1.61 |
|  | age>55 | 0.20 | 0.03 | 0.19 | 0.03 | 0.20 | 0.01 | 0.18 | 0.01 | 0.20 | 1.75 | 0.17 | 2.13 |

TG triglycerides; HDL, high-density lipoprotein; LDL, low-density lipoprotein; HbA1c, hemoglobin A1c; CRP, C-reactive Protein; SBP, systolic blood pressure; DBP, diastolic blood pressure; BMI, body mass index; WC, waist circumference; WHtR, waist-to-height ratio; CVD, cardiovascular disease

*log-transformed

Models were adjusted for age, sex, and smoking
