# Supplementary material for: Self-reported and measured anthropometric variables in association with cardiometabolic markers: A Danish cohort study
Source: PLoS One. 2023 Jul 27;18(7):e0279795. doi: 10.1371/journal.pone.0279795 (PMC10374072; doi:10.1371/journal.pone.0279795)
Supplement: S9 Table — (DOCX) [file pone.0279795.s009.docx]

S9 Table. ROC curve comparing measured and self-reported anthropometric indices with cardiovascular risk factors

| **Cardiovascular Disease Risk Factors** | | **Measured BMI** | | | **Self-reported BMI** | | | **Measured WC** | | | **Self-reported WC** | | | **Measure WHtR** | | | **Self-reported WHtR** | | |
| --- | --- | --- | --- | --- | --- | --- | --- | --- | --- | --- | --- | --- | --- | --- | --- | --- | --- | --- | --- |
|  |  |  |  |  |  |  |  |  |  |  |  |  |  |  |  |  |  |  |  |
|  |  | ROC | 95% CI | | ROC | 95% CI | | ROC | 95% CI | | ROC | 95% CI | | ROC | 95% CI | | ROC | 95% CI | |
| Hypertension | Model1 | 0.68 | 0.67 | 0.69 | 0.67 | 0.67 | 0.68 | 0.71 | 0.70 | 0.72 | 0.69 | 0.69 | 0.70 | 0.70 | 0.70 | 0.71 | 0.69 | 0.68 | 0.69 |
|  | Model2 | 0.76 | 0.76 | 0.77 | 0.76 | 0.76 | 0.77 | 0.75 | 0.75 | 0.76 | 0.74 | 0.74 | 0.75 | 0.76 | 0.75 | 0.76 | 0.75 | 0.74 | 0.75 |
| Dyslipidemia | Model1 | 0.74 | 0.73 | 0.75 | 0.73 | 0.73 | 0.74 | 0.78 | 0.77 | 0.79 | 0.76 | 0.76 | 0.77 | 0.76 | 0.75 | 0.76 | 0.74 | 0.73 | 0.75 |
|  | Model2 | 0.77 | 0.77 | 0.78 | 0.77 | 0.76 | 0.77 | 0.79 | 0.78 | 0.79 | 0.77 | 0.76 | 0.78 | 0.79 | 0.78 | 0.80 | 0.78 | 0.77 | 0.78 |
| Pre-diabetes | Model1 | 0.79 | 0.77 | 0.82 | 0.79 | 0.77 | 0.81 | 0.82 | 0.80 | 0.84 | 0.80 | 0.77 | 0.82 | 0.83 | 0.80 | 0.85 | 0.79 | 0.77 | 0.82 |
|  | Model2 | 0.84 | 0.82 | 0.86 | 0.84 | 0.82 | 0.86 | 0.84 | 0.82 | 0.86 | 0.83 | 0.81 | 0.85 | 0.85 | 0.83 | 0.87 | 0.83 | 0.81 | 0.85 |

BMI, body mass index; WC, waist circumference; WHtR, waist-to-height ratio; ROC, receiver operating curve

Model1, crude model;

Model 2, adjusted for age, sex, and smoking;
