# Supplementary material for: Self-reported and measured anthropometric variables in association with cardiometabolic markers: A Danish cohort study
Source: PLoS One. 2023 Jul 27;18(7):e0279795. doi: 10.1371/journal.pone.0279795 (PMC10374072; doi:10.1371/journal.pone.0279795)
Supplement: S10 Table — (DOCX) [file pone.0279795.s010.docx]

S10 Table. Correlation between self-reported and measured anthropometrics stratified by visiting time

| **Correlation** | **Questionnaire before visiting study center** | **Visiting study center before questionnaire** | **On the same day** |
| --- | --- | --- | --- |
| N(%) | 36,620 (92.7%) | 1,912 (4.8%) | 982 (2.5%) |
| Height | 0.98 | 0.99 | 0.98 |
| Weight | 0.99 | 0.99 | 0.99 |
| WC | 0.88 | 0.93 | 0.95 |
