# Supplementary material for: Self-reported and measured anthropometric variables in association with cardiometabolic markers: A Danish cohort study
Source: PLoS One. 2023 Jul 27;18(7):e0279795. doi: 10.1371/journal.pone.0279795 (PMC10374072; doi:10.1371/journal.pone.0279795)
Supplement: Attachment — Submitted filename: rebuttal letter.docx [file pone.0279795.s011.docx]

Journal Requirements:

When submitting your revision, we need you to address these additional requirements.

1. Please ensure that your manuscript meets PLOS ONE's style requirements, including those for file naming. The PLOS ONE style templates can be found at 
<https://journals.plos.org/plosone/s/file?id=wjVg/PLOSOne_formatting_sample_main_body.pdf> and 
<https://journals.plos.org/plosone/s/file?id=ba62/PLOSOne_formatting_sample_title_authors_affiliations.pdf>

Re: We have edited the manuscript to meet the style requirements.

2. Please provide additional details regarding participant consent. In the ethics statement in the Methods and online submission information, please ensure that you have specified what type you obtained (for instance, written or verbal, and if verbal, how it was documented and witnessed). If your study included minors, state whether you obtained consent from parents or guardians. If the need for consent was waived by the ethics committee, please include this information.

3. Please include your full ethics statement in the ‘Methods’ section of your manuscript file. In your statement, please include the full name of the IRB or ethics committee who approved or waived your study, as well as whether or not you obtained informed written or verbal consent. If consent was waived for your study, please include this information in your statement as well.

Re: For item 2 and 3, we have added the ethics approval part in the methods, please check Line 188-197 at page 9.

4. We note that the grant information you provided in the ‘Funding Information’ and ‘Financial Disclosure’ sections do not match.

When you resubmit, please ensure that you provide the correct grant numbers for the awards you received for your study in the ‘Funding Information’ section.

Re: We have edited this section, and included the amended statements within the cover letter.

5. Thank you for stating the following financial disclosure: 
" ‘The Diet, Cancer and Health –Next generations cohort’ was established with funding from the Danish Cancer Society, ‘Knæk Cancer 2012’ and ‘Den A.P Møllerske støttefond (grant no 10619)’.

No financial disclosures were reported by the authors of this paper."

Please state what role the funders took in the study.  If the funders had no role, please state: ""The funders had no role in study design, data collection and analysis, decision to publish, or preparation of the manuscript."" 
If this statement is not correct you must amend it as needed. 
Please include this amended Role of Funder statement in your cover letter; we will change the online submission form on your behalf.

Please use below funding statement:

"The Diet, Cancer and Health –Next generations cohort’ was established with funding from the Danish Cancer Society, ‘Knæk Cancer 2012’ and ‘Den A.P Møllerske støttefond (grant no 10619)’. This study was supported by an Aarhus University Research Foundation Starting Grant to C.C.D. (AUFF-F-2016-FLS-8-15).

The funders had no role in study design, data collection and analysis, decision to publish, or preparation of the manuscript. "

6. In your Data Availability statement, you have not specified where the minimal data set underlying the results described in your manuscript can be found. PLOS defines a study's minimal data set as the underlying data used to reach the conclusions drawn in the manuscript and any additional data required to replicate the reported study findings in their entirety. All PLOS journals require that the minimal data set be made fully available. For more information about our data policy, please see <http://journals.plos.org/plosone/s/data-availability>.

Upon re-submitting your revised manuscript, please upload your study’s minimal underlying data set as either Supporting Information files or to a stable, public repository and include the relevant URLs, DOIs, or accession numbers within your revised cover letter. For a list of acceptable repositories, please see <http://journals.plos.org/plosone/s/data-availability#loc-recommended-repositories>. Any potentially identifying patient information must be fully anonymized.

Important: If there are ethical or legal restrictions to sharing your data publicly, please explain these restrictions in detail. Please see our guidelines for more information on what we consider unacceptable restrictions to publicly sharing data: <http://journals.plos.org/plosone/s/data-availability#loc-unacceptable-data-access-restrictions>. Note that it is not acceptable for the authors to be the sole named individuals responsible for ensuring data access.

We will update your Data Availability statement to reflect the information you provide in your cover letter.

Re: We have updated data availability statement, please check line 386-390 at page 19.

7. Please review your reference list to ensure that it is complete and correct. If you have cited papers that have been retracted, please include the rationale for doing so in the manuscript text, or remove these references and replace them with relevant current references. Any changes to the reference list should be mentioned in the rebuttal letter that accompanies your revised manuscript. If you need to cite a retracted article, indicate the article’s retracted status in the References list and also include a citation and full reference for the retraction notice.
[Note: HTML markup is below. Please do not edit.]

Reviewers' comments:

Reviewer's Responses to Questions

**Comments to the Author**

1. Is the manuscript technically sound, and do the data support the conclusions?

The manuscript must describe a technically sound piece of scientific research with data that supports the conclusions. Experiments must have been conducted rigorously, with appropriate controls, replication, and sample sizes. The conclusions must be drawn appropriately based on the data presented.

Reviewer #1: Yes

Reviewer #2: Yes

2. Has the statistical analysis been performed appropriately and rigorously?

Reviewer #1: Yes

Reviewer #2: Yes

3. Have the authors made all data underlying the findings in their manuscript fully available?

The [PLOS Data policy](http://www.plosone.org/static/policies.action#sharing) requires authors to make all data underlying the findings described in their manuscript fully available without restriction, with rare exception (please refer to the Data Availability Statement in the manuscript PDF file). The data should be provided as part of the manuscript or its supporting information, or deposited to a public repository. For example, in addition to summary statistics, the data points behind means, medians and variance measures should be available. If there are restrictions on publicly sharing data—e.g. participant privacy or use of data from a third party—those must be specified.

Reviewer #1: Yes

Reviewer #2: No

4. Is the manuscript presented in an intelligible fashion and written in standard English?

PLOS ONE does not copyedit accepted manuscripts, so the language in submitted articles must be clear, correct, and unambiguous. Any typographical or grammatical errors should be corrected at revision, so please note any specific errors here.

Reviewer #1: Yes

Reviewer #2: Yes

5. Review Comments to the Author

Please use the space provided to explain your answers to the questions above. You may also include additional comments for the author, including concerns about dual publication, research ethics, or publication ethics. (Please upload your review as an attachment if it exceeds 20,000 characters)

**Self-reported and measured anthropometric variables in association with cardiometabolic markers: a Danish cohort study**

***************************************************************************
Dear Editors and Reviewers,

Thank you for the detailed comments and the insightful suggestions to improve our manuscript. We have edited the manuscript to incorporate changes that reflect the detailed suggestions you have kindly provided. To facilitate your review of our revisions, the following is a point-by-point response to the questions and comments.

************************************************************************

Reviewer #1: Dear Editor
I read interestingly the manuscript entitled “Self-reported and measured anthropometric variables in association with cardiometabolic markers: a Danish cohort study”. The study seems conducted carefully.

**- Please prepare a list of abbreviations at the beginning of the Meta DATA.**
Re: The abbreviations were provided at the end of the manuscript, please refer to line 357-375.

**Abstracts
-It is not clear in the abstract that why should we find an association between anthropometric indices and CVD diseases after we calibrated them, please justify it more clearly**

Re: Thank you for the comment. We have revised the abstract to emphasize the rational to study the association between anthropometric indices and CVD outcomes more clearly. A new sentence has been added in the abstract (Line 27-30).

*Line 27-30:* General obesity is a recognized risk factor for various metabolically related diseases, including hypertension, dyslipidemia, and pre-diabetes. In epidemiological studies, anthropometric variables such as height and weight are often self-reported.

**- Keywords: are these keywords are Mesh terms? Word that serves as a keyword, as to the meaning of that condition must be a Mesh term**
Re: Thank you for pointing this out, we have reviewed and updated the keywords to ensure they are Mesh terms.

Introduction
**- I am not sure about this claim of authors do they have any valid refrernce for it?
“In epidemiological studies, anthropometric variables are usually self-reported, especially in large study populations, as it is cost-effective and less burdensome compared to clinical measurements”.**

Re: Thank you for raising the concern. After further review, we have added several studies that have supported this claim, including a systematic review [1], and some large cohort studies[2, 3]. We have now included these references in our manuscript to strengthen the validity of our claim.

**Line 114: the authors started to discuss about blood pressure but they did not mention about it in the abstract**
Re: Thank you so much for pointing this out. We have revised the abstract to make it clear.

| Before | After |
| --- | --- |
| Self-reported and measured anthropometric variables, as well as cardiometabolic biomarkers, were obtained from participants aged aboved 18 years at recruitment to the Diet, Cancer, and Health-Next Generation Cohort in 2015-19 (N=39,514). | *Line 35-38:* A total of 39,514 participants aged above 18 years were included into the Diet, Cancer, and Health-Next Generation Cohort in 2015-19. Self-reported and measured anthropometric variables, blood pressure, and cardiometabolic biomarkers (HbA1c, lipid profiles, C-reactive protein and creatinine) were collected by standard procedures. |

Results
**There is no table that summarize demographic data of included participants, it would be better that authors report a table in this regard**
Re: **Appendix Table 1** is a description of demographic information of the participants, which was provided in supplementary information. To make it clear, we have moved this table to the main manuscript as Table 1, and the table numbers have been updated accordingly.

**Discussion
- The authors should list and shortly discuss the limitation of their study, for instance their the lack of evidence about justification of this research.**

Re: We appreciate your feedback and agree that it is important to acknowledge the limitation of the study.

In terms of the lack of evidence about the justification of our research, we would like to clarify that our study aimed to address the concern regarding misreporting in self-reported measures of anthropometry and its influence on estimates of associations between anthropometry and health outcomes. We have elaborated the justification of this study in the introduction (Line 57-72) and discussion (Line 286-296).

We acknowledge there are limitations of the study, for instance, the generalizability and representativeness of the cohort (Line 312-325).

**Reviewer #2: Although research idea is not novel and have been searched before . The authors made a great effort to re investigate the idea and correlated the results with cardiac biomarker. Regarding the sample size how did you calculate it and why didn't you mention it.**

Re: Thank you for the feedback and comments. This validation study was based on the Danish Diet, Cancer, and Health-Next Generation cohort (DCH-NG), with the purpose of examining whether self-reported anthropometrics are comparable with measured anthropometrics in terms of associated with cardiometabolic outcomes.

The DCH-NG is a population based cohort study. The sample size was calculated based on the number of cancer cases and other health outcomes expected to develop since 2011 as well as the magnitude of the relative risk to be detected, under the scenario with an estimated response rate of 30%. The sample size was determined to achieve a power of 80% and a significance level of 5%. The sample size calculation was based on the expected effect size and the variability of the target variables in the population. For details, please refer to the DCH-NG cohort description[4].

6. PLOS authors have the option to publish the peer review history of their article ([what does this mean?](https://journals.plos.org/plosone/s/editorial-and-peer-review-process#loc-peer-review-history)). If published, this will include your full peer review and any attached files.


If you choose “no”, your identity will remain anonymous but your review may still be made public.


**Do you want your identity to be public for this peer review?** For information about this choice, including consent withdrawal, please see our [Privacy Policy](https://www.plos.org/privacy-policy).

Reviewer #1: No

Reviewer #2: No

**Reference**

1.	Maukonen M, Männistö S, Tolonen H. A comparison of measured versus self-reported anthropometrics for assessing obesity in adults: a literature review. Scandinavian journal of public health. 2018;46(5):565-79.

2.	Hodge JM, Shah R, McCullough ML, Gapstur SM, Patel AV. Validation of self-reported height and weight in a large, nationwide cohort of US adults. PloS one. 2020;15(4):e0231229.

3.	Lipsky LM, Haynie DL, Hill C, Nansel TR, Li K, Liu D, et al. Accuracy of self-reported height, weight, and BMI over time in emerging adults. American journal of preventive medicine. 2019;56(6):860-8.

4.	Petersen KE, Halkjær J, Loft S, Tjønneland A, Olsen A. Cohort profile and representativeness of participants in the Diet, Cancer and Health—Next Generations cohort study. European journal of epidemiology. 2022:1-11.
